# Effects of dupilumab on quality of life in patients with atopic dermatitis: a systematic review and meta-analysis of randomized controlled trials

**DOI:** 10.3389/fphar.2025.1587977

**Published:** 2025-06-10

**Authors:** Yunqing Sun, Haibo Liu, Yingjun Gao, Hong Sang, Qingtao Kong

**Affiliations:** ^1^ Department of Dermatology, Jinling Hospital, Affiliated Hospital of Medical School, Nanjing University, Nanjing, China; ^2^ Department of Dermatology, Jinling Hospital, Nanjing Medical University, Nanjing, China

**Keywords:** atopic dermatitis, dupilumab, quality of life, meta-analysis, systematic review

## Abstract

**Objective:**

To evaluate the impact of dupilumab treatment on the quality of life, psychological factors and clinical symptoms, in patients with moderate-to-severe atopic dermatitis.

**Methods:**

A comprehensive search was conducted in PubMed, Web of Science, Embase, and Cochrane Library. Data coverage extends until April 2024. The study exclusively encompassed randomized controlled trials. The primary outcome was the quality of life of patients, with subgroup analyses conducted. Revman 5.4 and Stata 15.1 software were used for the analyses.

**Results:**

This study included 17 studies with a total of 6,665 participants. To evaluate quality of life, six instruments were used: the Dermatology Life Quality Index (DLQI), the Quality of Life Index for Atopic Dermatitis (QoLIAD), the Children’s Dermatology Life Quality Index (CDLQI), the Infant’s Dermatitis Quality of Life Index (IDQoL), the Dermatitis Family Impact (DFI), and the EuroQol-5 Dimension (EQ-5D). The QoLIAD is specifically designed for atopic dermatitis. The results demonstrated that dupilumab significantly improved DLQI/QoLIAD scores in adult patients (SMD = −0.64, 95% CI [-0.84, −0.45], p < 0.00001), the CDLQI/IDQoL scores in children/adolescents (SMD = −0.73, 95% CI [-0.84, −0.63], p < 0.00001), family quality of life measured by DFI (SMD = −0.97, 95% CI [-1.20, −0.75], p < 0.00001), and EQ-5D scores (SMD = 0.64, 95% CI [0.46, 0.82], p < 0.00001). The study also reported significant improvements in sleep-related metrics, total score on the Hospital Anxiety and Depression Scale (HADS), HADS anxiety scale, and HADS depression score. Additional improvements were observed in the Patient-Oriented Eczema Measure (POEM), pruritus Numeric Rating Scale (NRS), Eczema Area and Severity Index (EASI), SCORing Atopic Dermatitis (SCORAD), percent Body Surface Area (BSA), Global Individual Sign Score (GISS), and Investigator’s Global Assessment (IGA) response.

**Conclusion:**

Our study demonstrates that dupilumab significantly enhances patients’ quality of life and alleviates symptoms. This meta-analysis utilizes various assessment tools to evaluate quality of life, synthesizing data from recent clinical trials. It builds on prior research to provide a comprehensive analysis of dupilumab’s effects and overall efficacy. The aim of this study is to offer evidence-based insights to improve the clinical application of dupilumab.

**Systematic Review Registration:**

https://www.crd.york.ac.uk/PROSPERO/.

## 1 Introduction

Atopic dermatitis (AD) is a commonly found inflammatory skin condition, impacting approximately 20% of children and nearly 10% of adults in high-income regions (Laughter et al., 2020). It is primarily characterized by xerosis, severe itching, and persistent eczematous lesions (Langan et al., 2020). The burden of disability-adjusted life-years (DALYs) attributed to AD surpasses that of all other skin diseases, positioning it as the 15th most prevalent non-fatal disease globally (Laughter et al., 2020). AD greatly affects the quality of life for both patients and their caregivers. Patients often experience severe pruritus with a chronic and recurrent course, which can severely disrupt sleep, learning, work, and social interactions. In severe cases, patients may also develop psychological symptoms such as anxiety and depression (Girolomoni et al., 2021; Lewis-Jones and Finlay, 1995; Silverberg et al., 2018). The management of AD remains challenging due to limited responses to existing therapies. For mild to moderate AD, first-line therapy includes topical anti-inflammatory medications like corticosteroids and calcineurin inhibitors. However, patients with more severe AD who do not achieve sufficient relief from topical treatments require systemic therapy (Bieber, 2022). There are many traditional treatment methods for AD, but these therapies have limited efficacy or adverse effects in some moderate to severe cases, failing to meet clinical needs. With advancing understanding of the pathophysiology of AD, a series of biologics and targeted therapies have been developed. Dupilumab is currently recognized as an effective biologic for the management of AD.

Dupilumab is a human monoclonal antibody designed to specifically target the interleukin-4 receptor α (IL-4Rα). It disrupts the interaction between IL-4 and IL-13 with IL-4Rα, thus inhibiting Th2-mediated inflammatory responses (Beck et al., 2014; Berdyshev et al., 2022). An interventional study conducted in 2022 demonstrated that dupilumab improves skin barrier integrity in individuals suffering from moderate-to-severe AD, as indicated by decreased transepidermal water loss (TEWL) and changes in the lipid composition of the skin (Berdyshev et al., 2022). Clinical trials have shown that dupilumab offers marked improvements in clinical symptoms and patient-reported outcomes for moderate to severe AD compared to placebo, with consistent long-term efficacy and a strong safety profile (Simpson et al., 2016a; Blauvelt et al., 2017; Deleuran et al., 2020; Beck et al., 2022). A prospective cohort study spanning up to 5 years revealed that dupilumab provided rapid and enduring benefits in patient-reported outcomes across both adult and pediatric cohorts, confirming its safety and therapeutic efficacy (Zhang et al., 2024). Multiple real-world practice studies have yielded results consistent with clinical trials through long-term follow-ups, reflecting the potential value of dupilumab in treating AD in clinical settings (Ariëns et al., 2021; Lasek et al., 2022; Strober et al., 2022).

Currently, meta-analyses have confirmed the effectiveness and safety of dupilumab in managing AD. (Koskeridis et al., 2022; Wu and Wang, 2022) This meta-analysis utilized diverse instruments to evaluate patient quality of life, incorporating the latest clinical trials. It builds on earlier studies while providing a comprehensive analysis of how dupilumab affects patient quality of life and its efficacy. The study seeks to offer evidence-based insights to enhance the clinical use of dupilumab.

## 2 Methods

### 2.1 Protocol and study registration

This meta-analysis has been registered in the International Prospective Register of Systematic Reviews (PROSPERO) under registration number CRD42024544226. We conducted this study in accordance with the Preferred Reporting Items for Systematic Reviews and Meta-Analyses (PRISMA) (Liberati et al., 2009).

### 2.2 Search methods

An extensive search was carried out using PubMed, Web of Science, Embase, and the Cochrane Library, covering the period from the inception of these databases until 15 April 2024, to locate randomized controlled trials (RCTs) that evaluate the effectiveness of dupilumab in treating AD. The search employed both Medical Subject Headings (MeSH) and free-text terms. The English keywords searched encompassed “Dupilumab,” “Atopic Dermatitis,” and “Quality of Life,” without language restrictions applied. Additionally, we completed a manual examination of the references from the studies found to uncover additional pertinent trials. Comprehensive search methodologies can be found in Supplementary Table S1.

### 2.3 Selection criteria

#### 2.3.1 Inclusion

(a) Population: patients were diagnosed with chronic, moderate-to-severe atopic dermatitis (Eichenfield et al., 2014), as characterized by an Investigator’s Global Assessment (IGA) score ≥3, Eczema Area and Severity Index (EASI) score ≥12, involvement of ≥10% of body surface area, and inadequate response to topical AD treatments.
(b) Intervention: administration of dupilumab alone or with topical corticosteroids (TCS), regardless of the dose, frequency, or duration.
(c) Comparison: the use of other medications, excluding dupilumab, either as monotherapy or combined with TCS.
(d) Outcomes: the primary outcome assessed was quality of life, while secondary outcomes included psychological impact and efficacy indicators.
(e) Study design: randomized controlled trial.

#### 2.3.2 Exclusion

(a) Patients with atopic hand and foot dermatitis.
(b) Studies without available data can be extracted;
(c) Case reports, case series, and observational studies;
(d) Non-original studies (letters, reviews, editorials);
(e) Duplicate publication.

### 2.4 Data extraction

Data from every article were extracted separately by two researchers, with any conflicts were settled through discussion with a third researcher to achieve consensus. The data extracted encompassed the following:

(a) Publication Details: First author, and year of publication.
(b) Study Characteristics: Country and duration of follow-up.
(c) Participant Information: Number of patients included, treatment regimens, age, sex.
(d) Interventions: Dosage, frequency, administration route, and concurrent use of topical corticosteroids.
(e) Outcome Measures: Primary and secondary outcome.

### 2.5 Critical appraisal

Two researchers evaluated the quality of the studies included in the analysis using the Cochrane Risk of Bias Assessment Tool independently (Higgins et al., 2011). They cross-verified their evaluations. The assessment comprised seven domains: the random sequence generation; allocation concealment; blinding of outcome participants and personnel; blinding of outcome assessment; incomplete outcome data; selective reporting and other bias (Higgins et al., 2011). The methodological quality of the studies was classified into three levels: “high risk,” “low risk,” and “unclear.” (Higgins et al., 2011)

### 2.6 Statistical analysis

Reference management was conducted using EndNote 21, while data organization was performed using Excel. Statistical analyses were carried out using RevMan 5.4 and Stata 15.1.

The analysis of categorical variables, the effect measure used was the relative risk (RR), whereas the standardized mean difference (SMD) was utilized for continuous outcomes. The evaluation of heterogeneity incorporated a 95% confidence interval (CI) in conjunction with the I^2^ statistic. The analysis was carried out utilizing a random-effects model, where the significance threshold was set at α = 0.05. Sensitivity analyses included the sequential exclusion of individual studies. To evaluate publication bias, both funnel plots and Egger’s test were employed. Subgroup analyses were performed to examine the impact of dupilumab treatment regimen, concurrent use of TCS, patient age, follow-up duration, and control group interventions on quality of life measures, aiming to evaluate result stability and identify potential sources of heterogeneity.

## 3 Results

### 3.1 Research and selection

**FIGURE 1:**
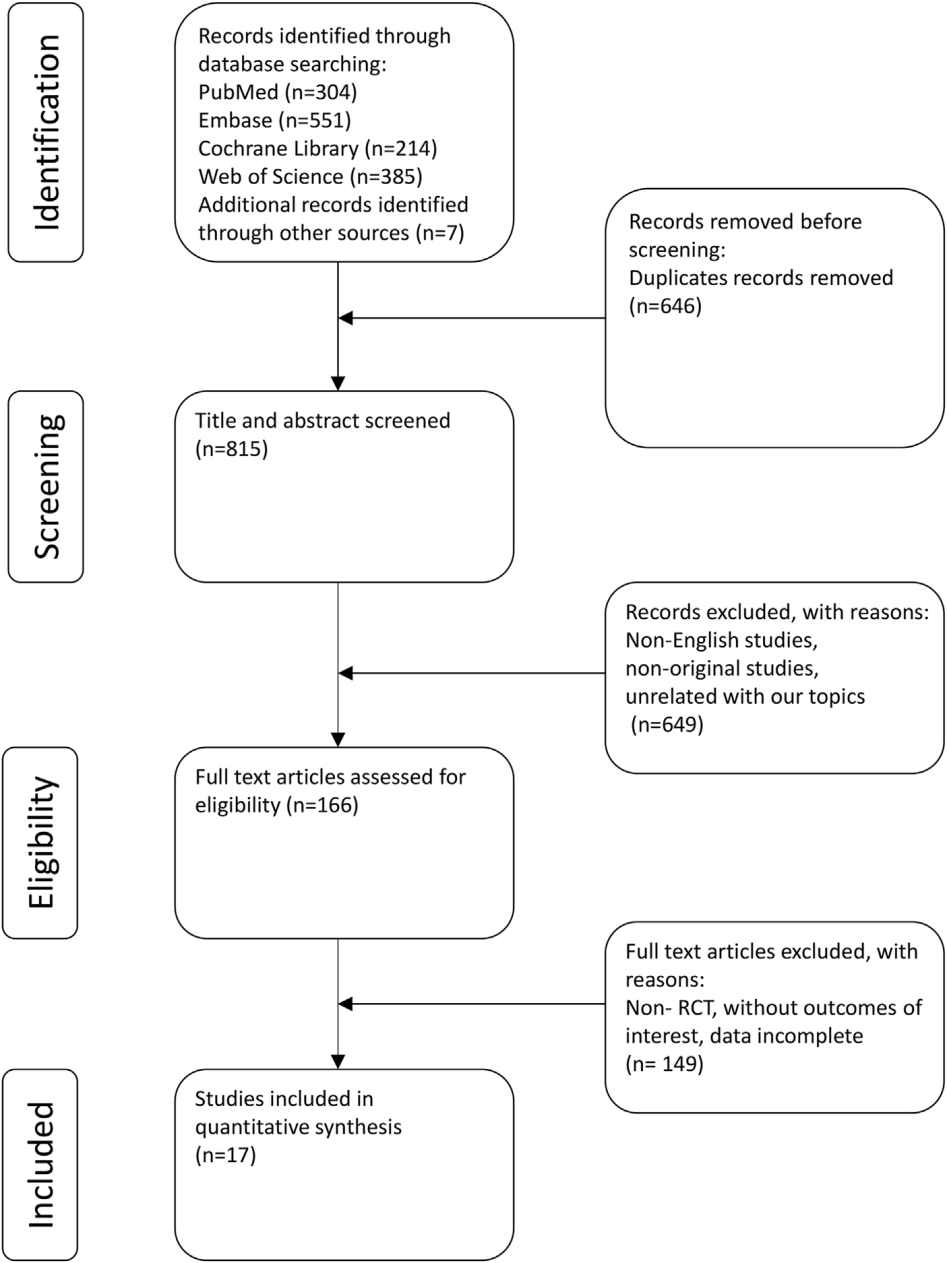
PRISMA flow diagram.

The strategy employed for the literature search and its corresponding outcomes are depicted in Figure 1. In total, 1,454 records were identified: 304 from PubMed, 385 from Web of Science, 551 from Embase, and 214 from the Cochrane Library. Additionally, manual reference list checks of included studies identified 7 more potentially eligible records. Following the literature screening, a final selection was made that included 13 clinical trials (including 17 studies) for analysis (Simpson et al., 2016a; Blauvelt et al., 2017; Bieber et al., 2021; Cork et al., 2024; de Bruin‐Weller et al., 2018; Merola et al., 2023; Paller et al., 2020a; Paller et al., 2024; Paller et al., 2020b; Paller et al., 2022; Reich et al., 2022; Siegfried et al., 2023; Simpson et al., 2016b; Simpson et al., 2020; Thaçi et al., 2016; Tsianakas et al., 2018; Zhao et al., 2022).

### 3.2 Study characteristics

Table 1 summarizes the baseline characteristics of the 17 studies examined. All were randomized controlled trials. 10 studies (10 trials) focused on adults, while 7 studies (3 trials) involved participants aged 6 months to 18 years, including both children and adolescents. 7 studies (4 trials) used concomitant topical corticosteroids, while 10 studies (9 trials) employed monotherapy. 2 studies used Abrocitinib as the control treatment. The follow-up periods varied from 12 to 52 weeks. In total, these studies enrolled 6,665 participants, with 3,910 participants allocated to the intervention group and 2,755 to the control group.

**TABLE 1:** Characteristics of included studies.

Study ID	Country	Participants	Intervention	Comparator	Follow-up (weeks)	Comment
Bieber 2021	Australia and countries across North and South America, Europe, and Asia	I/C: 242/131; Sex, male: 44.6%/58.8%; Mean age (years): 37.1/37.4 I/C: 242/226; Sex, male: 44.6%/50.4%; Mean age (years): 37.1/38.8 I/C: 242/238; Sex, male: 44.6%/46.0%; Mean age (years): 37.1/37.3	Dupilumab 300 mg q2w Dupilumab 300 mg q2w Dupilumab 300 mg q2w	Placebo Abrocitinib 200 mg/d Abrocitinib 100 mg/d	12	None
Blauvelt 2017	Australia, Canada, Czech Republic, Hungary, Italy, Japan, the Netherlands, New Zealand, Poland, Romania, South Korea, Spain, the United Kingdom, and the United States	I/C: 106/315; Sex, male: 58%/61%; Mean age (years): 36.70/37.35 I/C: 319/315; Sex, male: 60%/61%; Mean age (years): 35.05/34.70	Dupilumab 300 mg q2w + TCS Dupilumab 300 mg qw + TCS	Placebo + TCS Placebo + TCS	52	None
Cork 2024	Canada, the United States	I/C: 60/76; Sex, male: 58.3%/69.7%; Mean age (years): 4.0/3.8	Dupilumab 200/300 mg q4w + TCS	Placebo + TCS	16	*post hoc* analyses
De Bruin-Weller 2018	Austria, Belgium, Germany, Ireland, Netherlands, Poland, Russian Federation, Slovakia, Spain, the United Kingdom	I/C: 107/108; Sex, male: 60.7%/63.0%; Mean age (years): 36.59/38.56 I/C: 110/108; Sex, male: 60.0%/63.0%; Mean age (years): 38.35/38.56	Dupilumab 300 mg q2w + TCS Dupilumab 300 mg qw + TCS	Placebo + TCS Placebo + TCS	16	None
Merola 2023	Australia, France, Germany, Israel, Italy, Spain, Switzerland, the United Arab Emirates, the United Kingdom and the United States	I/C: 127/61; Sex, male: 48.0%/49.2%; Mean age (years):36.2/34.5	Dupilumab 300 mg q2w	Placebo	12	None
Paller 2020 Ⅰ	Canada, Czech Republic, Germany, Poland, the United Kingdom, and the United States	I/C: 122/123; Sex, male: 46.7%/49.6%; Mean age (years): 8.5/8.3 I/C: 122/123; Sex, male: 53.3%/49.6%; Mean age (years): 8.5/8.5	Dupilumab 300 mg q4w + TCS Dupilumab 100/200 mg q4w + TCS	Placebo + TCS Placebo + TCS	16	None
Paller 2020 Ⅱ	Canada, the United States	I/C: 69/83; Sex, male: 64%/61%; Mean age (years): 14.3/14.4 I/C: 62/83; Sex, male: 48%/61%; Mean age (years): 14.6/14.4	Dupilumab 300 mg q4w Dupilumab 200/300 mg q2w	Placebo Placebo	16	*post hoc* analyses
Paller 2022	Canada, the United States	I/C: 83/79; Sex, male: 53%/70%; Mean age (years): 3.9/3.8	Dupilumab 200/300 mg q4w + TCS	Placebo + TCS	16	None
Paller 2024	Canada, the United States	I/C: 63/62; Sex, male: 58.7%/67.7%; Mean age (years): 3.9/3.9	Dupilumab 200/300 mg q4w + TCS	Placebo + TCS	16	pre-specified subgroup analysis
Reich 2022	Australia, Bulgaria, Canada, Chile, Finland, Germany, Hungary, Italy, Latvia, Poland, Slovakia, South Korea, Spain, Taiwan, and the United States	I/C: 365/362; Sex, male: 56%/53%; Mean age (mean): 35.5/36.6	Dupilumab 300 mg q2w	Abrocitinib 200 mg qd	26	None
Siegfried 2023	Canada, Czech Republic, Germany, Poland, the United Kingdom, and the United States	I/C: 82/109; Sex, male: 46.3%/47.7%; Mean age (years): 8.5/8.4 I/C: 36/109; Sex, male: 61.1%/47.7%; Mean age (years): 9.7/8.4	Dupilumab 300 mg q4w + TCS Dupilumab 200 mg q2w + TCS	Placebo + TCS Placebo + TCS	16	*post hoc* analyses
Simpson 2016 Ⅰ	Canada, Czech Republic, Germany, Hungary, Japan, Poland, the United States	I/C: 63/61; Sex, male: 68%/66%; Mean age (years): 36.2/37.2 I/C: 64/61; Sex, male: 64%/66%; Mean age (years): 39.4/37.2 I/C: 61/61; Sex, male: 59%/66%; Mean age (years): 35.8/37.2 I/C: 65/61; Sex, male: 62%/66%; Mean age (years): 36.8/37.2 I/C: 65/61; Sex, male: 52%/66%; Mean age (years): 36.6/37.2	Dupilumab 300 mg qw Dupilumab 300 mg q2w Dupilumab 200 mg q2w Dupilumab 300 mg q4w Dupilumab 100 mg q4w	Placebo Placebo Placebo Placebo Placebo	16	*post hoc* analyses
Simpson 2016 Ⅱ	North America, Europe, and Asia	I/C: 457/460; Sex, male: 58.4%/54.3%; Mean age (years): 36.70/37.35 I/C: 462/460; Sex, male: 60.8%/54.3%; Mean age (years): 37.05/37.35	Dupilumab 300 mg q2w Dupilumab 300 mg qw	Placebo Placebo	16	None
Simpson 2020	Canada, the United States	I/C: 84/85; Sex, male: 61.9%/62.4%; Mean age (years): 14.4/14.5 I/C: 82/85; Sex, male: 52.4%/62.4%; Mean age (years): 14.5/14.5	Dupilumab 300 mg q4w Dupilumab 200/300 mg q2w	Placebo Placebo	16	None
Thaçi 2016	Canada, Czech Republic, Germany, Hungary, Japan, Poland, the United States	I/C: 63/61; Sex, male: 68%/66%; Mean age (years): 36.2/37.2 I/C: 64/61; Sex, male: 64%/66%; Mean age (years): 39.4/37.2 I/C: 61/61; Sex, male: 59%/66%; Mean age (years): 35.8/37.2 I/C: 65/61; Sex, male: 62%/66%; Mean age (years): 36.8/37.2 I/C: 65/61; Sex, male: 52%/66%; Mean age (years): 36.6/37.2	Dupilumab 300 mg qw Dupilumab 300 mg q2w Dupilumab 200 mg q2w Dupilumab 300 mg q4w Dupilumab 100 mg q4w	Placebo Placebo Placebo Placebo Placebo	16	None
Tsianakas 2018	Czechia, France, Germany, Hungary, Poland	I/C: 32/32; Sex, male: 59%/53%; Mean age (years): 37.3/40.7	Dupilumab 300 mg qw	Placebo	12	None
Zhao 2021	China	I/C: 82/83; Sex, male: 70.7%/72.3%; Mean age (years): 29.06/27.41	Dupilumab 300 mg q2w	Placebo	16	None

I/C: intervention/control group.

### 3.3 Bias risk assessment

The studies incorporated in this analysis underwent rigorous evaluation utilizing the Cochrane risk of bias assessment tool. Among the studies evaluated, sixteen were determined to have a low risk of bias, whereas one study suggested the potential presence of other biases. A comprehensive summary of the quality assessment is illustrated in Supplementary Figure S1.

### 3.4 Meta analysis results

All studies assessed the QoL of patients. Six different tools were utilized. For adult patients, nine studies used the Dermatology Life Quality Index (DLQI), and one study used the Quality of Life Index for Atopic Dermatitis (QoLIAD), both of which associate higher scores with more significant quality of life impairment. Seven studies focusing on children and adolescents used the Children’s Dermatology Life Quality Index (CDLQI) and the Infants’ Dermatitis Quality of Life Index (IDQoL). Three studies employed the Dermatology Family Index (DFI), and two studies used the EuroQol Quality of Life 5-Dimension (EQ-5D). We also performed a separate analysis focusing on sleep disturbances and anxiety and depression symptoms as reported across the included studies.

### 3.5 Primary outcomes

#### 3.5.1 Change in health-related quality of life (HRQoL)

**FIGURE 2:**
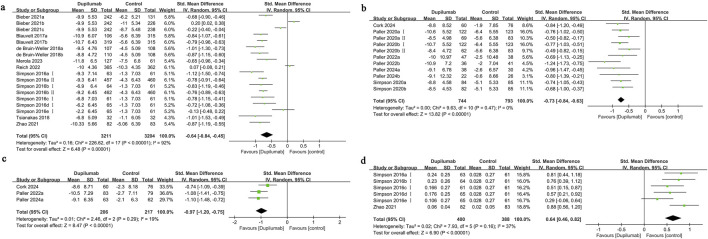
Forest plot of quality of life-related indicators. **(a)** HRQoL; **(b)** CDLQI or IDQoL; **(c)** DFI; **(d)** EQ-5D.

Eighteen cohorts from nine studies were assessed regarding HRQoL among adults. Of these studies, eight implemented DLQI, whereas one utilized QoLIAD, a questionnaire specifically developed for adult patients affected by AD. The findings demonstrated that dupilumab significantly improved the quality of life for individuals experiencing moderate-to-severe AD, in comparison to the control group (SMD = −0.64, 95% CI] [-0.84, −0.45], I^2^ = 92%, p < 0.00001, Figure 2a), indicating substantial heterogeneity.

The funnel plot displayed no significant imbalance in the HRQoL results (Supplementary Figure S2), and the assessment using Egger’s test revealed no substantial publication bias (Egger’s test P = 0.276). Subgroup analysis indicated that the heterogeneity might be attributed to the dupilumab dosing regimen, concomitant use of TCS, and the selection of control group medication. When analyzing the variations based on the control group medication type, it was revealed that individuals treated with dupilumab experienced a significant enhancement in quality of life when compared to those given a placebo (SMD = −0.78, 95% CI [-0.86, −0.70], I^2^ = 37%, p < 0.00001). Conversely, when abrocitinib was used as the control, the results indicated no statistically meaningful difference in quality of life outcomes between the dupilumab and abrocitinib groups (SMD = 0.02, 95% CI [-0.21, 0.25], I^2^ = 82%, p = 0.87) (Supplementary Table S2).

#### 3.5.2 Change in Children’s Dermatology Life Quality Index (CDLQI) or infants’ dermatology quality of Life Index (IDQoL)

Eleven cohorts from six studies the quality of life outcomes for children and adolescents receiving treatment were evaluated. The findings indicated that pediatric patients receiving dupilumab exhibited a significantly greater enhancement in quality of life compared to those in the control group (SMD = −0.73, 95% CI [-0.84, −0.63], I^2^ = 0%, p < 0.00001, Figure 2b). The funnel plot demonstrated no significant asymmetry for this measure (Supplementary Figure S2), and Egger’s test suggested no substantial evidence of publication bias (Egger’s test P = 0.250).

#### 3.5.3 Change in dermatitis family index (DFI)

Three studies examined family quality of life, showing that improvements were more significant in the group receiving dupilumab than in the control group (SMD = −0.97, 95% CI [-1.20, −0.75], I^2^ = 19%, p < 0.00001, Figure 2c). The funnel plot demonstrated no significant asymmetry in the DFI results (Supplementary Figure S2), and Egger’s test revealed no signs of publication bias (Egger’s test P = 0.960).

#### 3.5.4 Change in EuroQol quality of life 5-dimension (EQ-5D)

Six cohort studies from two research projects reported on the EQ-5D outcomes. The improvement in utility scores for patients treated with dupilumab was significantly greater than that for patients given a placebo treatment (SMD = 0.64, 95% CI [0.46, 0.82], I^2^ = 37%, p < 0.00001, Figure 2d). The funnel plot demonstrated no significant asymmetry in the EQ-5D results (Supplementary Figure S2), and Egger’s test found no evidence of publication bias (Egger’s test P = 0.519).

### 3.6 Secondary outcomes

#### 3.6.1 Change in sleep

Eighteen cohorts from eight studies reported changes in sleep quality before and after treatment. A comparison between the dupilumab and control groups revealed that patients receiving dupilumab experienced significant improvements in sleep quality. (SMD = −0.64, 95% CI [-0.81, −0.47], I^2^ = 84%, p < 0.00001, Supplementary Figure S3).

The funnel plot indicated significant asymmetry in these results (Supplementary Figure S2), and Egger’s test indicated substantial publication bias (Egger’s test P = 0.004).

Subgroup analysis suggested that the heterogeneity might be attributed to factors such as the dosing regimen, concomitant use of TCS, patient age, treatment duration, and medications used in the control group. The comparison between the intervention and control groups showed no notable differences when using either a 300 mg weekly or 100 mg every 4 weeks dupilumab regimen (p = 0.78, p = 0.29, respectively). Additionally, when the control group medication was abrocitinib, the variations between the dupilumab and abrocitinib groups did not reach statistical significance (p = 0.69).

#### 3.6.2 Change in Hospital Anxiety depression scale (HADS)

In the evaluation of the overall HADS score, a total of fifteen cohorts from six different studies were analyzed. The findings revealed that individuals receiving dupilumab demonstrated a more significant improvement in HADS scores when compared to the control group (SMD = −0.43, 95% CI [-0.51, −0.35], I^2^ = 32%, p < 0.00001, Supplementary Figure S3).

Three studies comprising ten cohorts separately assessed anxiety symptoms. The findings demonstrated that the improvement in anxiety symptoms was more pronounced in the group treated with dupilumab compared to the control group (SMD = −0.25, 95% CI [-0.40, −0.11], I^2^ = 62%, p = 0.0006, Supplementary Figure S3). The subgroup analysis pointed out that the considerable heterogeneity may stem from variations in the dupilumab dosing regimen, the age of patients, and duration of treatment (Supplementary Table S2).

Additionally, three studies with ten cohorts evaluated depressive symptoms, revealing that the reduction in depressive symptoms was more pronounced in the dupilumab group than in the control group (SMD = −0.25, 95% CI [-0.44, −0.07], I^2^ = 77%, p = 0.007, Supplementary Figure S3). The significant heterogeneity observed may be related to the dupilumab dosing regimen, patient age, treatment duration, and medication used in the control group (Supplementary Table S2).

Funnel plots indicated no significant asymmetry in the HADS total score and HADS depression scale results (Supplementary Figure S2), and Egger’s test did not reveal significant publication bias (Egger’s test P = 0.950 and P = 0.059, respectively). However, the funnel plot for the HADS anxiety scale exhibited significant asymmetry (Supplementary Figure S2), and Egger’s test indicated significant publication bias (Egger’s test P = 0.023).

#### 3.6.3 Change in patient oriented eczema measure (POEM)

Data from twenty-two cohorts across twelve studies provided insights into alterations in the POEM scores when compared to baseline measurements. The analysis showed that the intervention group exhibited a significantly larger reduction in POEM scores compared to the control group (SMD = −0.90, 95% CI [-1.07, −0.72], I^2^ = 90%, p < 0.00001, Supplementary Figure S4). A review of the funnel plot indicated notable asymmetry (Supplementary Figure S2), and Egger’s test demonstrated a significant publication bias (Egger’s test P = 0.045). Subgroup analyses found significant statistical differences in all subgroups except when the dosing regimen was 100 mg every 4 weeks and the control drug was Abrocitinib, where no significant difference was observed (Supplementary Table S2).

#### 3.6.4 Change in pruritus numerical rating scale (NRS)

Thirteen studies comprising twenty-three cohorts reported changes in Pruritus NRS scores from baseline. Patients treated with dupilumab experienced significantly greater improvement in pruritus assessment compared to those in the control group (SMD = −0.87, 95% CI [-0.94, −0.79], I^2^ = 33%, p < 0.00001, Supplementary Figure S4). The funnel plot showed no significant asymmetry (Supplementary Figure S2), and Egger’s test suggested the absence of considerable publication bias (Egger’s test P = 0.811).

#### 3.6.5 Investigator Global Assessment (IGA) response

Eleven studies comprising twenty-two cohorts reported the IGA response. Patients treated with dupilumab showed a significantly higher proportion of achieving an IGA score of 0 or 1, as well as a 2-point or greater improvement from baseline, compared to those receiving other treatments (RR = 3.45, 95% CI [2.34, 5.09], I^2^ = 94%, p < 0.00001, Supplementary Figure S4). The funnel plot indicated significant asymmetry in these results (Supplementary Figure S2), and Egger’s test revealed substantial publication bias (Egger’s test P = 0.000).

#### 3.6.6 Change in Eczema Area and Severity Index (EASI) scores

Fifteen studies comprising twenty-eight cohorts reported changes in EASI scores from baseline. The change in EASI scores was greater in the intervention group compared to the control group (SMD = −0.90, 95% CI [-1.05, −0.75], I^2^ = 89%, p < 0.00001, Supplementary Figure S4). The funnel plot indicated significant asymmetry in these results (Supplementary Figure S2), and Egger’s test revealed substantial publication bias (Egger’s test P = 0.012).

#### 3.6.7 Change in SCORing atopic dermatitis (SCORAD) score

Thirteen studies comprising twenty-three cohorts reported changes in SCORAD scores from the starting point. The enhancement observed in SCORAD scores was greater in the intervention group relative to the control group (SMD = −0.99, 95% CI [-1.17, −0.80], I^2^ = 91%, p < 0.00001, Supplementary Figure S4). Analysis of the funnel plot showed notable asymmetry in these findings (Supplementary Figure S2), while Egger’s test pointed to considerable publication bias (Egger’s test P = 0.021).

#### 3.6.8 Change in body surface area (BSA)

Twelve studies comprising twenty-two cohorts reported changes in BSA from baseline. The variation in BSA from baseline was notably more pronounced in patients belonging to the intervention group when contrasted with the control group (SMD = −0.77, 95% CI [-0.96, −0.58], I^2^ = 92%, p < 0.00001, Supplementary Figure S4). The funnel plot indicated significant asymmetry in these results (Supplementary Figure S2), while Egger’s test uncovered significant publication bias (Egger’s test P = 0.004).

#### 3.6.9 Change in global individual signs score (GISS)

Six studies comprising ten cohorts reported changes in GISS scores from baseline. Those who underwent the intervention showed a greater enhancement in GISS scores when compared to individuals in the control group (SMD = −0.83, 95% CI [-0.90, −0.76], I^2^ = 0%, p < 0.00001, Supplementary Figure S4). The analysis of the funnel plot displayed notable asymmetry in the findings (Supplementary Figure S2), while Egger’s test suggested a considerable presence of publication bias (Egger’s test P = 0.007).

### 3.7 Sensitivity analysis

This study conducted sensitivity analyses on all outcome measures using a leave-one-out method. The findings revealed that the removal of any individual study had no significant impact on the variability of the indicators, implying that these measures demonstrate considerable stability (Supplementary Figure S5).

## 4 Discussion

Atopic dermatitis develops through a multifactorial interplay of genetic predisposition, skin barrier impairment, immune system dysregulation, environmental factors, and microbial colonization, with Th2 pathway activation serving as a central feature of the disease. Dupilumab, a monoclonal IgG4 antibody, selectively binds to the IL-4Rα, disrupting IL-4 and IL-13 signaling pathways, which reduces the Th2 immune response (Facheris et al., 2023). Additionally, Dupilumab has been shown to decrease Th2 biomarkers in the blood, demonstrating its therapeutic efficacy not only in treating cutaneous lesions but also in alleviating symptoms in unaffected skin and addressing other systemic manifestations of AD. (Rothenberg-Lausell et al., 2024)

This study provides evidence that dupilumab substantially enhances the quality of life for individuals of both adult and pediatric age groups suffering from moderate to severe AD. Subjective symptoms, including sleep disturbances, pruritus, psychological health, and self-assessed severity of eczema, showed notable improvement. Furthermore, all clinical indices, such as IGA, EASI, SCORAD, BSA, and GISS, showed marked improvement.

Our research presents fresh perspectives that extend prior meta-analyses on the application of dupilumab for AD treatment. The meta-analysis performed by Koskeridis et al. comprised 12 RCTs and performed subgroup analyses for both adult and pediatric/adolescent populations, confirming the efficacy and safety of dupilumab across all age groups. However, the limited number of studies involving children and adolescents may not provide sufficiently reliable conclusions (Koskeridis et al., 2022). In 2021, Wu et al. executed a meta-analysis that included 11 RCTs, which demonstrated that dupilumab significantly improved symptoms and overall quality of life for patients suffering from moderate to severe AD, while maintaining a favorable safety profile (Wu and Wang, 2022). An observational prospective cohort study by Miniotti et al. further demonstrated that dupilumab rapidly alleviated AD symptoms and had a sustained positive impact on both the quality of life and mental health (Miniotti et al., 2022).

Earlier research has mainly concentrated on the influence of medications in alleviating disease symptoms and improving clinical indicators. In contrast, this study offers a more detailed analysis of how dupilumab affects quality of life. Furthermore, this study incorporates recent randomized controlled trials published within the past 3 years, which feature a larger sample size, thereby increasing the statistical robustness of the findings. Our assessment of patients is multifaceted, confirming and extending the findings of previous meta-analyses. In assessing patients’ quality of life, this meta-analysis utilizes six different instruments (DLQI, QoLIAD, CDLQI, IDQoL, DFI, EQ-5D), providing a multidimensional substantiation of the significant quality of life improvements observed in AD patients receiving dupilumab.

To further interpret our findings, we examined representative studies from both adult and pediatric populations. For example, Simpson et al. (2016) focused on adult patients and reported substantial improvements in HRQoL outcomes following dupilumab treatment, which aligns with our subgroup findings (Simpson et al., 2016a). Similarly, Paller et al. (2022) investigated pediatric patients also reported significant improvements in HRQoL outcomes (Paller et al., 2022). These representative studies illustrate that the overall benefits of dupilumab are observed across different age groups, despite variations in study design and patient characteristics. The consistency of these results underscores the broad applicability of dupilumab’s effects.

A subgroup analysis based on different dupilumab dosing regimens revealed that when administered at 100 mg every 4 weeks, no statistically significant difference was observed between the dupilumab group and the control group regarding DLQI and EQ-5D outcomes. This subgroup included only one study with a treatment follow-up duration of 16 weeks, suggesting that this dosage may not achieve and maintain a stable response following the initial loading dose in a short-term treatment. An analysis of subgroups based on the use of control group medications showed no significant differences across outcome measures when comparing abrocitinib to dupilumab. Abrocitinib operates as an oral Janus kinase 1 (JAK1) inhibitor, influencing several cytokines, including IL-4, IL-13, and IFN-γ, which are key players in the pathophysiology of AD. By blocking the JAK-STAT signaling pathway, abrocitinib effectively reduces the inflammatory response associated with this condition (Gooderham et al., 2019). In contrast, dupilumab is a biologic agent targeting an extracellular receptor subunit, offering more precise targeting compared to immunosuppressants and oral JAK inhibitors (Butala et al., 2023).

In addition to its demonstrated efficacy in improving quality-of-life outcomes, the safety profile of dupilumab is an important consideration for clinical use. Common adverse events associated with dupilumab treatment include injection-site reactions and conjunctivitis, which have been frequently reported in clinical trials (Blauvelt et al., 2017; Simpson et al., 2016b). These adverse events are generally mild to moderate and manageable through supportive care, dose adjustment, or temporary discontinuation. Early recognition and proactive management are crucial to maintaining treatment adherence and optimizing outcomes.

The progression of AD is primarily influenced by an immune response dominated by Th2 cells, which involves crucial cytokines like IL-4 and IL-13 (Simpson et al., 2016b). These cytokines can bind to receptors on peripheral nerves in the skin, triggering pruritus (Tominaga and Takamori, 2022). The pathways of IL-4 and IL-13, mediated by Th2 cells, activate the JAK signaling pathway, subsequently promoting the release of additional cytokines, including IL-31, a potent inducer of itching (Dubin et al., 2021). Dupilumab inhibits critical pathogenic processes in AD by modulating immune responses, altering the disease-specific epidermal barrier pathologies, and controlling disease progression (Guttman-Yassky et al., 2019). Pruritus is the most prevalent and distressing symptom for AD patients, leading to a cycle of scratching that worsens skin lesions (Langan et al., 2020; Mack and Kim, 2018; Evers et al., 2019) and are associated with stress, anxiety, and depression (Evers et al., 2019). When the disease is inadequately controlled, patients often experience varying degrees of sleep disturbances (Cameron et al., 2024). AD can also impair social functioning (Coelho et al., 2024; Capec et al., 2022), and parents of pediatric patients may suffer psychological distress, reducing family quality of life (Yang et al., 2019; Drucker et al., 2017). Due to the rapid and significant improvement in symptoms provided by dupilumab, both pruritus and rash severity are alleviated, thereby enhancing patient quality of life.

This research is subject to several limitations that warrant consideration. Firstly, this meta-analysis concentrated exclusively on RCTs, and a frequent drawback of many RCTs is their small sample sizes, potentially resulting in small sample size effects. Furthermore, the strict control conditions of RCTs result in high homogeneity, which may not reflect the diversity of the general population and may not fully reflect the experiences of patients in everyday clinical settings. Additionally, the majority of participants in these studies are from Europe and North America, with fewer Asian patients, resulting in regional representation imbalance. Further research is necessary to confirm whether the improvements in quality of life observed with this drug can be generalized. There is significant heterogeneity in measures such as the DLQI, sleep quality, HADS anxiety and depression subscales, EASI, SCORAD, BSA, POEM, and IGA, which may affect the interpretation of results. However, this study identified potential sources of heterogeneity through subgroup analysis, which is one of its strengths.

## 5 Conclusion

Our study indicates that dupilumab significantly improves patients’ quality of life and alleviates symptoms. Further research and clinical applications may reveal more about the prolonged impacts and possible side effects of this medication in different patient populations, thereby optimizing individualized treatment plans and maximizing its clinical benefits.

## Data Availability

The original contributions presented in the study are included in the article/Supplementary Material, further inquiries can be directed to the corresponding authors.
