# Supplementary material for: Effects of dupilumab on quality of life in patients with atopic dermatitis: a systematic review and meta-analysis of randomized controlled trials
Source: Front Pharmacol. 2025 Jun 10;16:1587977. doi: 10.3389/fphar.2025.1587977 (PMC12185402; doi:10.3389/fphar.2025.1587977)
Supplement: Supplementary file 1 [file Supplementaryfile1.docx]

**Supplementary materials**

**TableS1.** Search strategies of database

| **Pubmed-304** |
| --- |
| ((("dupilumab" [Supplementary Concept]) OR ((((((SAR231893) OR (SAR-231893)) OR (Dupixent)) OR (REGN668)) OR (REGN-668)) OR (Dupilumab))) AND (("Dermatitis, Atopic"[Mesh]) OR (((((((((Atopic Dermatitis) OR (Neurodermatitis, Atopic)) OR (Atopic Neurodermatitis)) OR (Neurodermatitis, Disseminated)) OR (Disseminated Neurodermatitis)) OR (Eczema, Atopic)) OR (Atopic Eczema)) OR (Eczema, Infantile)) OR (Infantile Eczema)))) AND (("Quality of Life"[Mesh]) OR (((((Life Quality) OR (Health-Related Quality Of Life)) OR (Health Related Quality Of Life)) OR (HRQOL)) OR (QOL))) |
|  |
| **Embase-551** |
| **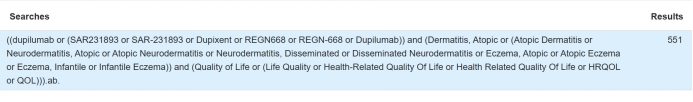** |
|  |
| **Cochrane-214** |
| 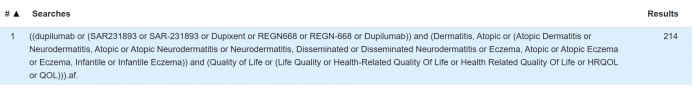 |
|  |
| **WOS-385** |
| (((dupilumab) OR ((((((SAR231893) OR (SAR-231893)) OR (Dupixent)) OR (REGN668)) OR (REGN-668)) OR (Dupilumab))) AND ((Dermatitis, Atopic) OR (((((((((Atopic Dermatitis) OR (Neurodermatitis, Atopic)) OR (Atopic Neurodermatitis)) OR (Neurodermatitis, Disseminated)) OR (Disseminated Neurodermatitis)) OR (Eczema, Atopic)) OR (Atopic Eczema)) OR (Eczema, Infantile)) OR (Infantile Eczema)))) AND ((Quality of Life) OR (((((Life Quality) OR (Health-Related Quality Of Life)) OR (Health Related Quality Of Life)) OR (HRQOL)) OR (QOL))) (Topic) |

**TableS2.** Subgroup analysis of dupilumab treatment in patients with moderate to severe atopic dermatitis

| Subgroup | HRQoL | | | | CDLQI or IDQoL | | | | EQ-5D | | | |
| --- | --- | --- | --- | --- | --- | --- | --- | --- | --- | --- | --- | --- |
|  | Study | SMD [95%CI] | *P* value | *I*^2^ | Study | SMD [95%CI] | *P* value | *I*^2^ | Study | SMD [95%CI] | *P* value | *I*^2^ |
| ***Total*** | 18 | -0.64 [-0.84, -0.45] | <0.00001 | 92% | 11 | -0.73 [-0.84, -0.63] | <0.00001 | 0% | 6 | 0.64 [0.46, 0.82] | <0.00001 | 37% |
| ***Dupilumab dose & Frequency of Administration*** |  |  |  |  |  |  |  |  |  |  |  |  |
| 300mg qw | 5 | -0.81 [-0.91, -0.72] | <0.00001 | 0% | 0 | NA | NA | NA | 1 | 0.81 [0.44, 1.18] | <0.00001 | NA |
| 300mg q2w | 10 | -0.55 [-0.84, -0.26] | 0.0002 | 95% | 0 | NA | NA | NA | 2 | 0.83 [0.59, 1.07] | <0.00001 | 0% |
| 200mg q2w | 1 | -0.78 [-1.15, -0.41] | <0.00001 | NA | 0 | NA | NA | NA | 1 | 0.51 [0.15, 0.87] | 0.006 | NA |
| 300mg q4w | 1 | -0.72 [-1.08, -0.36] | <0.00001 | NA | 3 | -0.68 [-0.85, -0.51] | <0.00001 | 0% | 1 | 0.57 [0.21, 0.92] | 0.002 | NA |
| 100mg q4w | 1 | -0.13 [-0.48, 0.22] | 0.46 | NA | 0 | NA | NA | NA | 1 | 0.29 [-0.06, 0.64] | 0.11 | NA |
| ***Therapies*** |  |  |  |  |  |  |  |  |  |  |  |  |
| Dupilumab monotherapy | 14 | -0.57 [-0.80, -0.34] | <0.00001 | 93% | 4 | -0.61 [-0.77, -0.45] | <0.00001 | 0% | 6 | 0.64 [0.46, 0.82] | <0.00001 | 37% |
| Dupilumab plus TCS | 4 | -0.85 [-0.96, -0.74] | <0.00001 | 0% | 7 | -0.82 [-0.96,-0.69] | <0.00001 | 0% | 0 | NA | NA | NA |
| ***Age*** |  |  |  |  |  |  |  |  |  |  |  |  |
| ≥18 y | 18 | -0.64 [-0.84, -0.45] | <0.00001 | 92% | 0 | NA | NA | NA | 6 | 0.64 [0.46, 0.82] | <0.00001 | 37% |
| <18 y | 0 | NA | NA | NA | 11 | -0.73 [-0.84, -0.63] | <0.00001 | 0% | 0 | NA | NA | NA |
| ***Follow-up*** |  |  |  |  |  |  |  |  |  |  |  |  |
| ≥16 weeks | 13 | -0.72 [-0.93, -0.51] | <0.00001 | 91% | 11 | -0.73 [-0.84, -0.63] | <0.00001 | 0% | 6 | 0.64 [0.46, 0.82] | <0.00001 | 37% |
| <16 weeks | 5 | -0.44 [-0.83, -0.04] | 0.03 | 92% | 0 | NA | NA | NA | 0 | NA | NA | NA |
| ***Control group medication*** |  |  |  |  |  |  |  |  |  |  |  |  |
| Placebo | 15 | -0.78 [-0.86, -0.70] | <0.00001 | 37% | 11 | -0.73 [-0.84, -0.63] | <0.00001 | 0% | 6 | 0.64 [0.46, 0.82] | <0.00001 | 37% |
| Abrocitinib | 3 | 0.02 [-0.21, 0.25] | 0.87 | 82% | 0 | NA | NA | NA | 0 | NA | NA | NA |

**TableS2. (continue)** Subgroup analysis of dupilumab treatment in patients with moderate to severe atopic dermatitis

| Subgroup | HADS total score | | | | HADS anxiety scale | | | | HADS depression scale | | | |
| --- | --- | --- | --- | --- | --- | --- | --- | --- | --- | --- | --- | --- |
|  | Study | SMD [95%CI] | *P* value | *I*^2^ | Study | SMD [95%CI] | *P* value | *I*^2^ | Study | SMD [95%CI] | *P* value | *I*^2^ |
| ***Total*** | 15 | -0.43 [-0.51, -0.35] | <0.00001 | 32% | 10 | -0.25 [-0.40, -0.11] | <0.00001 | 0% | 10 | -0.25 [-0.44, -0.07] | 0.007 | 77% |
| ***Dupilumab dose & Frequency of Administration*** |  |  |  |  |  |  |  |  |  |  |  |  |
| 300mg qw | 4 | -0.47 [-0.63, -0.32] | <0.00001 | 57% | 1 | -0.57 [-0.93, -0.21] | 0.002 | NA | 1 | -0.78 [-1.15, -0.42] | <0.00001 | NA |
| 300mg q2w | 4 | -0.49 [-0.65, -0.32] | 0.0002 | 52% | 4 | -0.20 [-0.45, 0.04] | 0.11 | 80% | 4 | -0.20 [-0.50, 0.09] | 0.18 | 86% |
| 200mg q2w | 1 | -0.64 [-1.00, -0.27] | <0.00001 | NA | 1 | -0.48 [-0.84, -0.12] | 0.009 | NA | 1 | -0.01 [-0.36, 0.35] | 0.96 | NA |
| 300mg q4w | 3 | -0.36 [-0.54, -0.17] | <0.00001 | 0% | 2 | -0.18 [-0.41, 0.06] | 0.14 | 0% | 2 | -0.22 [-0.63, 0.19] | 0.29 | 67% |
| 100mg q4w | 1 | -0.38 [-0.73, -0.02] | 0.46 | NA | 1 | -0.31 [-0.66, 0.04] | 0.08 | NA | 1 | -0.35 [-0.70, 0.00] | 0.05 | NA |
| ***Therapies*** |  |  |  |  |  |  |  |  |  |  |  |  |
| Dupilumab monotherapy | 11 | -0.45 [-0.54, -0.36] | <0.00001 | 18% | 10 | -0.25 [-0.40, -0.11] | 0.0006 | 62% | 10 | -0.25 [-0.44, -0.07] | 0.007 | 77% |
| Dupilumab plus TCS | 4 | -0.41 [-0.58, -0.24] | <0.00001 | 57% | 0 | NA | NA | NA | 0 | NA | NA | NA |
| ***Age*** |  |  |  |  |  |  |  |  |  |  |  |  |
| ≥18 y | 11 | -0.47 [-0.55, -0.39] | <0.00001 | 31% | 8 | -0.29 [-0.46, -0.11] | 0.001 | 70% | 8 | -0.24 [-0.46, -0.02] | 0.03 | 81% |
| <18 y | 4 | -0.26 [-0.42, -0.11] | NA | 0% | 2 | -0.13 [-0.35, 0.10] | 0.28 | 0% | 2 | -0.32 [-0.55, -0.09] | 0.007 | 0% |
| ***Follow-up*** |  |  |  |  |  |  |  |  |  |  |  |  |
| ≥16 weeks | 15 | -0.43 [-0.51, -0.35] | <0.00001 | 91% | 7 | -0.34 [-0.48, -0.20] | <0.00001 | 17% | 7 | -0.34 [-0.48, -0.20] | <0.00001 | 17% |
| <16 weeks | 0 | NA | NA | NA | 3 | -0.11 [-0.35, 0.12] | 0.34 | 77% | 3 | -0.11 [-0.35, 0.12] | 0.34 | 77% |
| ***Control group medication*** |  |  |  |  |  |  |  |  |  |  |  |  |
| Placebo | 15 | -0.43 [-0.51, -0.35] | <0.00001 | 37% | 8 | -0.34 [-0.46, -0.23] | <0.00001 | 4% | 8 | -0.35 [-0.53, -0.17] | 0.0001 | 58% |
| Abrocitinib | 0 | NA | NA | NA | 2 | -0.00 [-0.14, 0.14] | 0.97 | 15% | 2 | 0.05 [-0.07, 0.18] | 0.4 | 0% |

**TableS2. (continue)** Subgroup analysis of dupilumab treatment in patients with moderate to severe atopic dermatitis

| Subgroup | Sleep | | | | Pruritus NRS | | | | POEM | | | |
| --- | --- | --- | --- | --- | --- | --- | --- | --- | --- | --- | --- | --- |
|  | Study | SMD [95%CI] | *P* value | *I*^2^ | Study | SMD [95%CI] | *P* value | *I*^2^ | Study | SMD [95%CI] | *P* value | *I*^2^ |
| ***Total*** | 18 | -0.64 [-0.81, -0.47] | <0.00001 | 84% | 23 | -0.87 [-0.94, -0.79] | <0.00001 | 33% | 22 | -0.90 [-1.07, -0.72] | <0.00001 | 90% |
| ***Dupilumab dose & Frequency of Administration*** |  |  |  |  |  |  |  |  |  |  |  |  |
| 300mg qw | 2 | -0.99 [-1.29, -0.69] | 0.78 | 0% | 4 | -1.00 [-1.26, -0.74] | <0.00001 | 78% | 4 | -1.03 [-1.26, -0.81] | <0.00001 | 78% |
| 300mg q2w | 5 | -0.49 [-0.87, -0.10] | 0.01 | 93% | 5 | -0.85 [-0.98, -0.73] | <0.00001 | 22% | 6 | -1.01 [-1.22, -0.80] | <0.00001 | 74% |
| 200mg q2w | 1 | -0.61 [-0.97, -0.24] | 0.001 | NA | 2 | -0.86 [-1.13, -0.59] | <0.00001 | 0% | 1 | -1.31 [-1.71, -0.92] | <0.00001 | NA |
| 300mg q4w | 4 | -0.63 [-0.80, -0.46] | <0.00001 | 13% | 5 | -0.83 [-0.97, -0.69] | <0.00001 | 0% | 4 | -0.87 [-1.17, -0.57] | <0.00001 | 73% |
| 100mg q4w | 1 | -0.19 [-0.54, 0.16] | 0.29 | NA | 1 | -0.42 [-0.77, -0.06] | 0.02 | NA | 1 | -0.31 [-0.66, 0.05] | 0.09 | NA |
| ***Therapies*** |  |  |  |  |  |  |  |  |  |  |  |  |
| Dupilumab monotherapy | 14 | -0.58 [-0.77, -0.38] | <0.00001 | 84% | 14 | -0.88 [-0.98, -0.77] | <0.00001 | 43% | 14 | -0.80 [-1.05, -0.56] | <0.00001 | 92% |
| Dupilumab plus TCS | 4 | -0.84 [-0.99, -0.70] | <0.00001 | 0% | 9 | -0.84 [-0.93, -0.74] | <0.00001 | 8% | 8 | -1.04 [-1.13, -0.95] | <0.00001 | 0% |
| ***Age*** |  |  |  |  |  |  |  |  |  |  |  |  |
| ≥18 y | 10 | -0.57 [-0.83, -0.31] | <0.00001 | 88% | 13 | -0.87 [-0.98, -0.76] | <0.00001 | 54% | 14 | -0.94 [-1.18, -0.70] | <0.00001 | 93% |
| <18 y | 8 | -0.73 [-0.84, -0.62] | <0.00001 | 3% | 10 | -0.87 [-0.97, -0.77] | <0.00001 | 0% | 8 | -0.83 [-1.03, -0.64] | <0.00001 | 67% |
| ***Follow-up*** |  |  |  |  |  |  |  |  |  |  |  |  |
| ≥16 weeks | 13 | -0.70 [-0.81, -0.58] | <0.00001 | 35% | 21 | -0.87 [-0.94, -0.80] | <0.00001 | 29% | 21 | -0.91 [-1.09, -0.73] | <0.00001 | 91% |
| <16 weeks | 5 | -0.52 [-0.92, -0.11] | 0.01 | 93% | 2 | -0.94 [-1.58, -0.29] | 0.004 | 77% | 1 | -0.65 [-0.96, -0.34] | <0.00001 | NA |
| ***Control group medication*** |  |  |  |  |  |  |  |  |  |  |  |  |
| Placebo | 16 | -0.72 [-0.82, -0.63] | <0.00001 | 28% | 23 | -0.87 [-0.94, -0.79] | <0.00001 | 33% | 21 | -0.94 [-1.06, -0.83] | <0.00001 | 73% |
| Abrocitinib | 2 | -0.04 [-0.24, 0.16] | 0.69 | 59% | 0 | NA | NA | NA | 1 | 0.06 [-0.08, 0.21] | 0.41 | NA |

HRQoL, health-related quality of life; CDLQI, Children's Dermatology Life Quality Index; IDQoL, Infants' Dermatology Quality of Life Index; DFI, Dermatitis Family Index; EQ-5D, EuroQol Quality of Life 5-Dimension; HADS, Hospital Anxiety Depression Scale; POEM, Patient Oriented Eczema Measure; NA, not available.


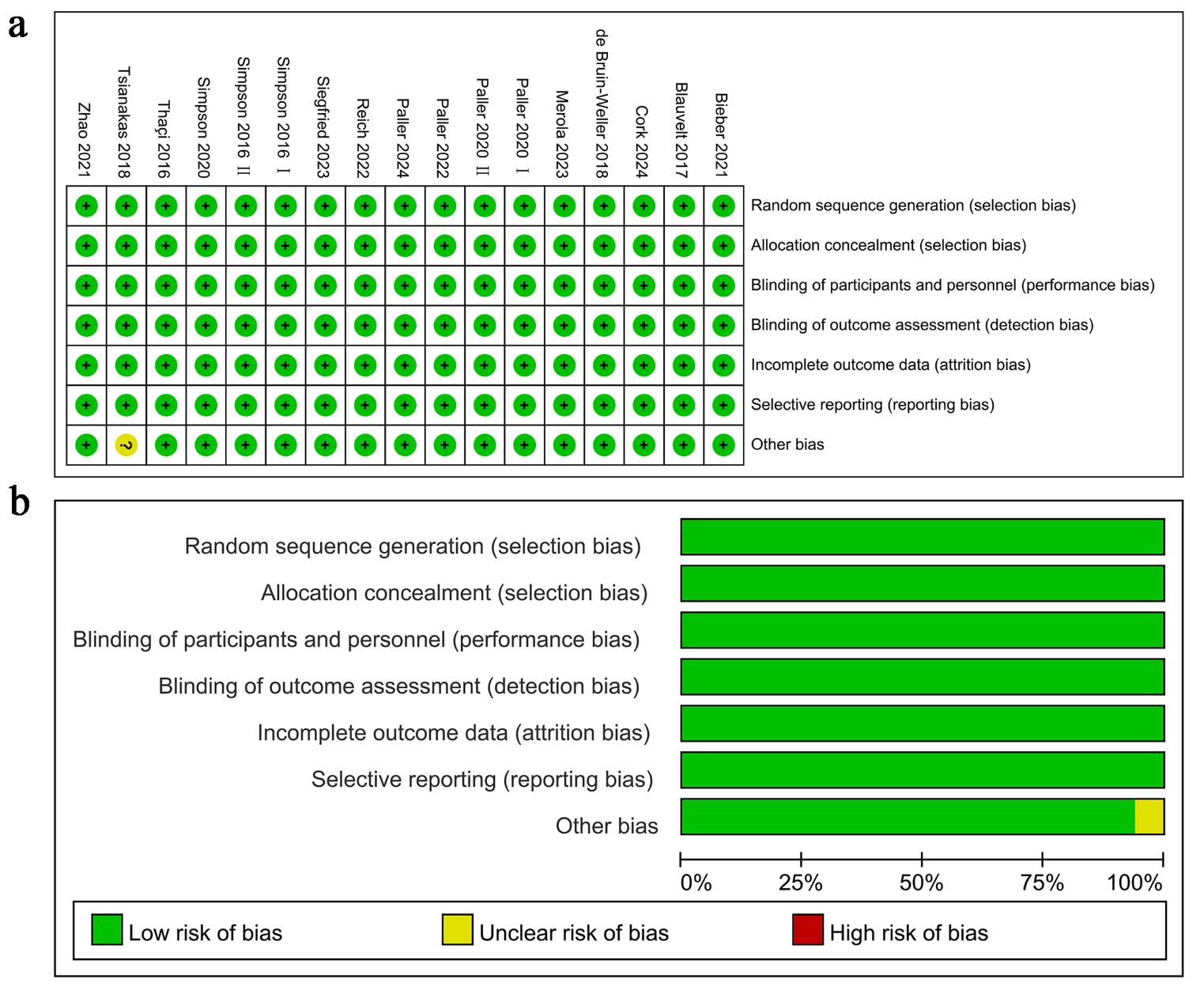


**FigureS1** (A) Risk of bias summary; (B) Risk of bias graph


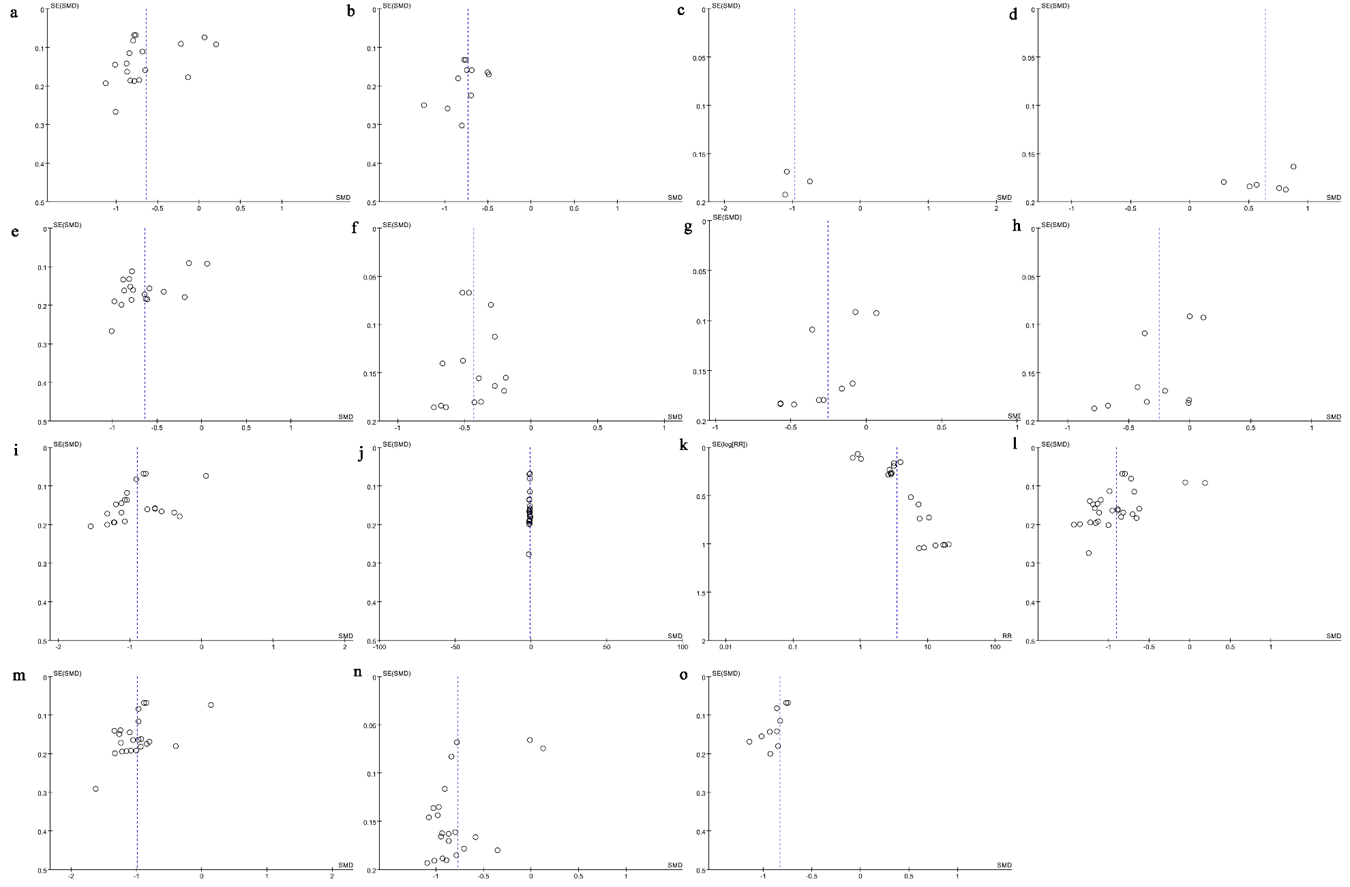


**FigureS2** Funnel plot. (a) HRQoL; (b) CDLQI/IDQoL; (c) DFI; (d) EQ-5D; (e) Sleep; (f) HADS total score; (g) HADS anxiety subscale; (h) HADS depression subscale; (i) POEM; (j) Pruritus NRS; (k) IGA response; (l) EASI; (m) SCORAD; (n) BSA; (o) GISS.


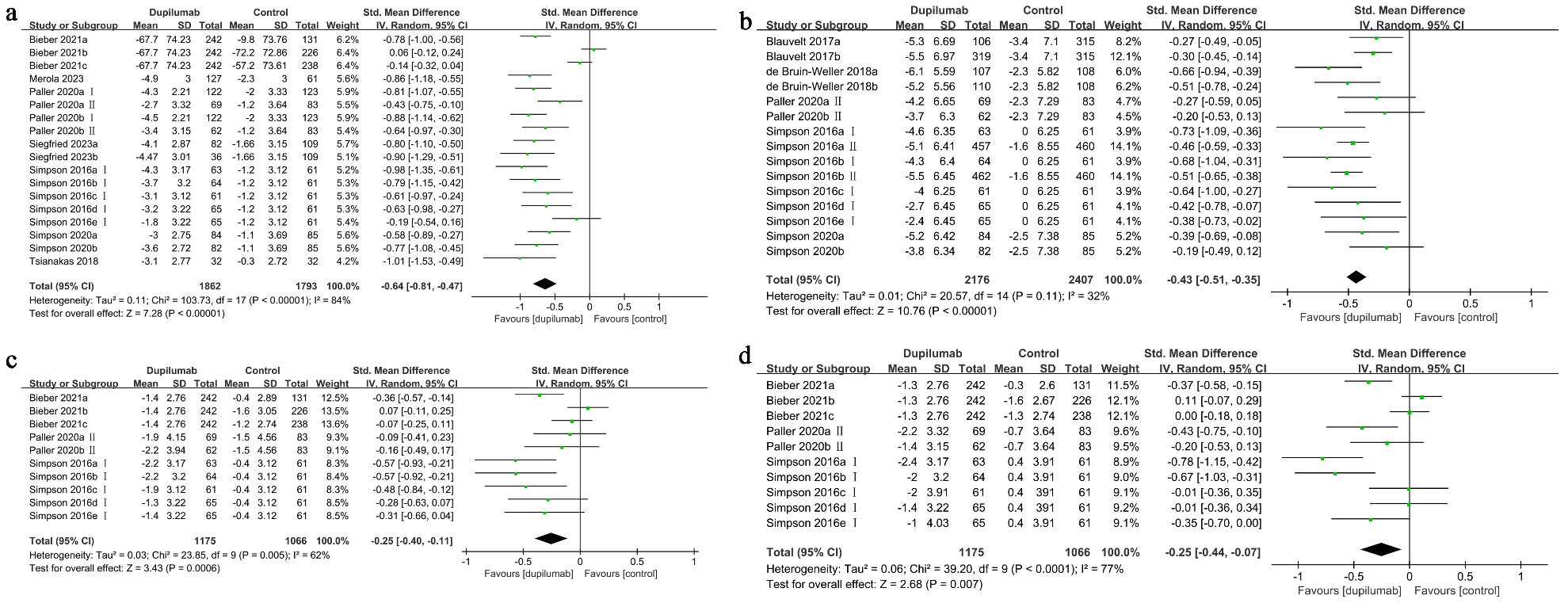


**FigureS3** Forest plot of sleep and psychological symptoms. (a) Sleep; (b) HADS total score; (c) HADS anxiety subscale; (d) HADS depression subscale.


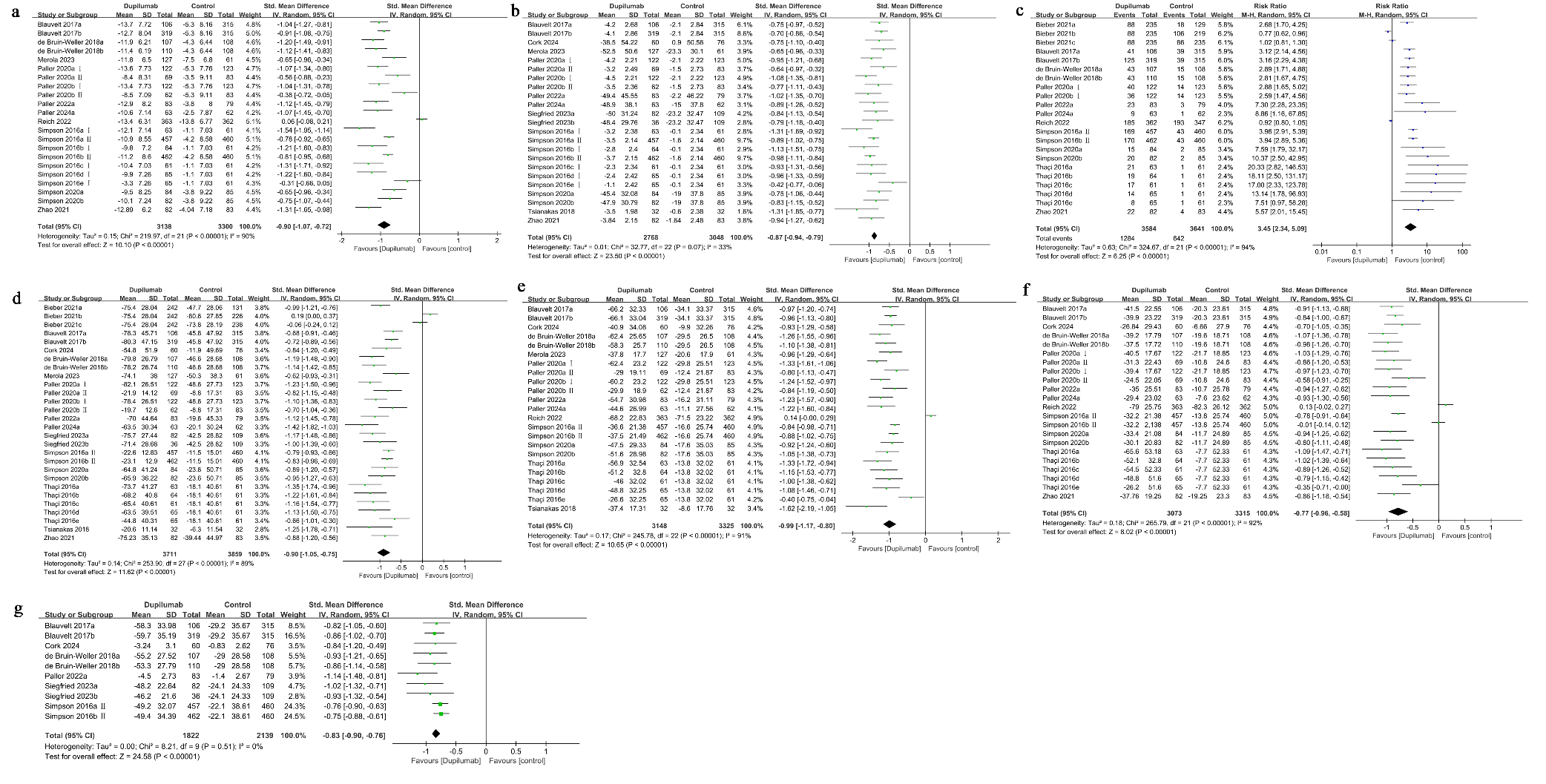


**FigureS4** Forest plot of clinical symptom-related indicators. (a) POEM; (b) Pruritus NRS; (c) IGA response; (d) EASI; (e) SCORAD; (f) BSA; (g) GISS.


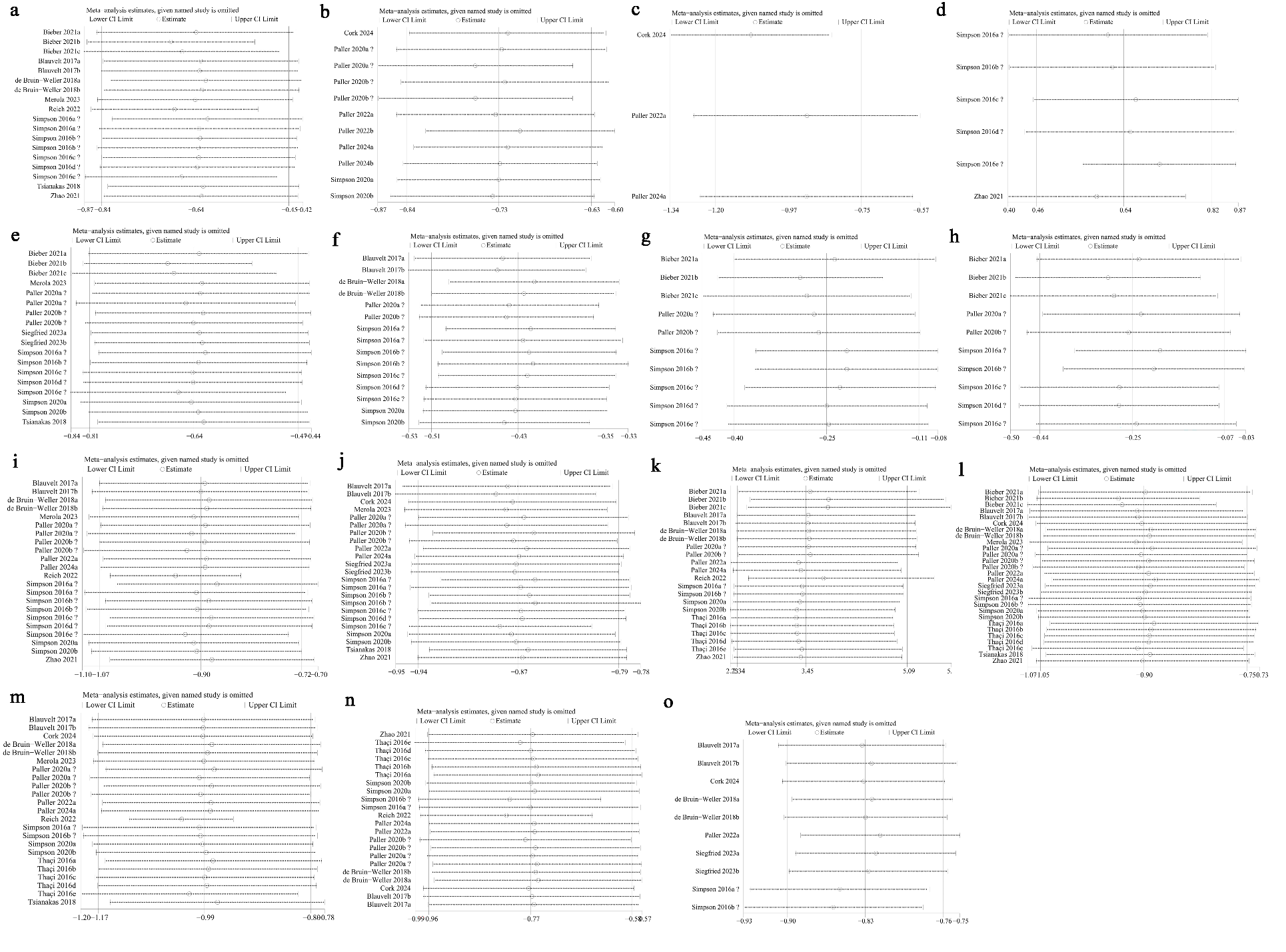


**FigureS5** sensitivity analysis. (a) HRQoL; (b) CDLQI/IDQoL; (c) DFI; (d) EQ-5D; (e) Sleep; (f) HADS total score; (g) HADS anxiety subscale; (h) HADS depression subscale; (i) POEM; (j) Pruritus NRS; (k) IGA response; (l) EASI; (m) SCORAD; (n) BSA; (o) GISS.
